# Silencing of METTL3 prevents the proliferation, migration, epithelial-mesenchymal transition, and renal fibrosis of high glucose-induced HK2 cells by mediating WISP1 in m6A-dependent manner

**DOI:** 10.18632/aging.205401

**Published:** 2024-01-29

**Authors:** Yuanzhen Chen, Ping Li, Mei Lin, Ying Jiang, Guiping Tan, Lianfang Huang, Dan Song

**Affiliations:** 1 Department of Nephrology, Shenzhen Guangming District People’s Hospital, Guangming, Shenzhen 518000, China

**Keywords:** diabetic nephropathy, high glucose, METTL3, WISP1, m6A modification

## Abstract

Diabetic nephropathy (DN) is one of the most serious complications in diabetic patients. And m6A modifications mediated by METTL3 are involved multiple biological processes. However, the specific function and mechanism of METTL3 in DN remains unclear. DN model mice were first established with streptozotocin, and WISP1 expression was confirmed by qRT-PCR. Then the influences of WISP1 or/and METTL3 on the proliferation, migration, and epithelial-mesenchymal transition (EMT) and fibrosis-related proteins of high glucose (HG)-induced HK2 cells or HK2 cells were tested through CCK-8, wound healing, and western blot. We first revealed that WISP1 was highly expressed in renal tissues of DN model mice and HG-induced HK2 cells. Functionally, WISP1 or METTL3 silencing could weaken the proliferation, migration, EMT, and fibrosis of HG-treated HK2 cells, and WISP1 or METTL3 overexpression could induce the proliferation, migration, EMT, and fibrosis of HK2 cells. Additionally, METTL3 silencing could decrease WISP1 m6A modification, and silencing of METTL3 also could notably suppress the biological functions of HG-induced HK2 cells by downregulating WISP1. Silencing of METTL3 prevents DN development process by decreasing WISP1 with m6A modification pattern. Therefore, we suggest that METTL3/WISP1 axis might be a novel therapeutic target for DN.

## INTRODUCTION

Diabetic nephropathy (DN) is the most frequent microvascular complication of diabetes mellitus, which can cause chronic kidney damage leading to glomerulopathy [1, 2]. DN is mainly characterized by progressive proteinuria and renal function decline, which eventually progresses to end-stage renal disease [3]. DN has multiple morphological changes, including podocyte apoptosis, thylakoid cell hypertrophy and proliferation, and epithelial mesenchymal transition (EMT) of renal tubular cells, etc. [4–6]. Among them, tubular cell EMT is the most familiar cause of renal function impairment [7]. Susceptibility factors for DN include disorders of metabolic mechanisms, genetics, hyperglycemia, inflammatory responses, hemodynamic alterations, and oxidative stress, etc. [8, 9]. Research showed that there is a “legacy effect” in diabetic patients, and epigenetic factors play a key role in this effect.

The methylation modification (m6A) occurring on the nitrogen atom at position 6 of adenine is the most familiar methylation modification in eukaryotic mRNA [10]. METTL3 is the core component of the m6A methyltransferase complex and is responsible for catalyzing m6A modifications in RNA [11]. METTL3 can play key regulatory roles at the post-transcriptional levels, including precursor mRNA splicing, export of mature mRNA, and mRNA stability regulation, thus affecting multiple biological processes [12]. In type 2 diabetes, glucose can regulate METTL3-mediated m6A modifications [13]. Recent studies also testified that METTL3-mediated m6A modification of TIMP2 can enhance podocyte injury in DN [14]; METTL3 can attenuate renal injury and interstitial fibrosis by enhancing NSD2 stability [15]. However, studies on the pathogenesis of METTL3 in DN are far from adequate. Further exploration of the genes that METTL3 may modify in DN has significant implications for DN therapy.

Renal fibrosis is a typical pathophysiological change that accompanies the progression of DN disease and is the main pathway leading to chronic kidney disease to end-stage renal failure [16]. And cellular EMT induced by renal tubular epithelial cell injury is thought to be a key factor in renal fibrosis [17]. The Wnt/β-catenin pathway is a key pathway regulating EMT in renal tubular epithelial cells [18]. Therefore, suppression of Wnt/β-catenin pathway attenuates EMT in renal tubular epithelial cells as a key mechanism to alleviate tubulointerstitial fibrosis in DN. WISP1 has been reported to be a downstream molecule of the Wnt/β-catenin pathway [19]. WISP1 can affect the growth and development of cells by influencing their survival, proliferation, migration, and differentiation, resulting in an inflammatory response [20, 21]. WISP1 can affect the proliferation and differentiation of kidney cells by affecting Wnt/β-catenin, which can lead to kidney disease [19]. However, the role of WISP1 in DN is not clear, and whether METTL3 can influence DN processes through modifying WISP1 in m6A-dependent manner has not been reported.

In our study, we first confirmed the change of WISP1 expression in DN model mice. We also verified the effects of WISP1 and METTL3 silencing or overexpression on the proliferation, migration, EMT and fibrosis of high glucose (HG)-induced HK2 cells or HK2 cells. Further, we explored the influence of METTL3 on WISP1 m6A modification and whether METTL3 could accelerate the DN process by changing WISP1 m6A modification. Through our current study, we can conclusively demonstrate that METTL3-mediated WISP1 might be the molecular target for DN therapy, which also provides a m6A modification pattern in DN development.

## RESULTS

### WISP1 was upregulated in renal tissues of DN model mice and HG-induced HK2 cells

**Figure 1:**
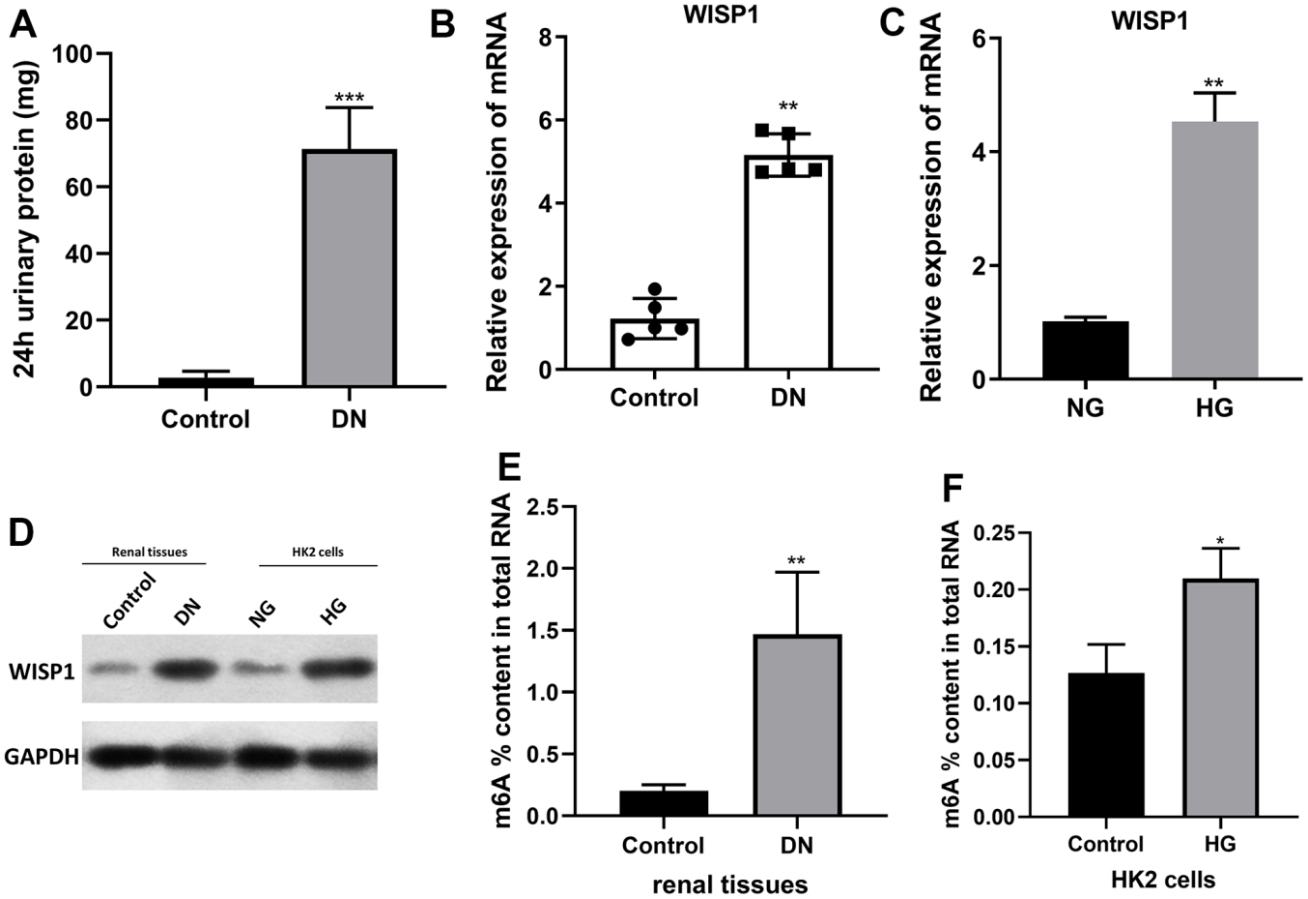
**WISP1 expression was highly expressed in DN model mice and HG-treated HK2 cells.** DN model mice were first established using streptozotocin. (**A**) 24-h urinary protein. (**B**) qRT-PCR analysis of WISP1 expression in kidney tissues which were removed from normal and DN mice (n=5). (**C**) QRT-PCR analysis of WISP1 expression in HG-treated HK2 cells. (**D**) The protein level expression of WISP1 in renal tissues and HK2 cells. (**E**) The total m6A RNA methylation context of total RNA between control and DN group. (**F**) The total m6A RNA methylation context of total RNA between control and HG group. *P < 0.05, **P < 0.01.

To explore possible key genes in DN development, we first constructed DN mouse model with 40mg/kg streptozotocin. The 24 h urinary albumin of DN mouse was significantly higher than that of control group (1A). That indicated successful diabetic nephropathy modeling. And qRT-PCR data denoted that the level of WISP1 in the DN group was notably increased relative to that in the control group (Figure 1B). Then HK2 cells were induced by HG. And the data revealed that the level of WISP1 in the HG group was obviously elevated compared with that in the NG group (Figure 1C). The expression of WISP1 in renal tissues and HK cells were also to be confirmed at protein levels (Figure 1D). Thus, this result presented that the upregulation of WISP1 might be associated with DN. The proportion (%) of m6A in total RNA was calculated. This data confirmed that the total levels of m6A methylation were higher in DN group than those in control group (Figure 1E). Similar results were also found in cell model. The proportion (%) of m6A in total RNA was increased significantly in HG group (Figure 1F).

### WISP1 overexpression promoted the proliferation, migration, EMT, and renal fibrosis of HG-induced HK2 cells

**Figure 2:**
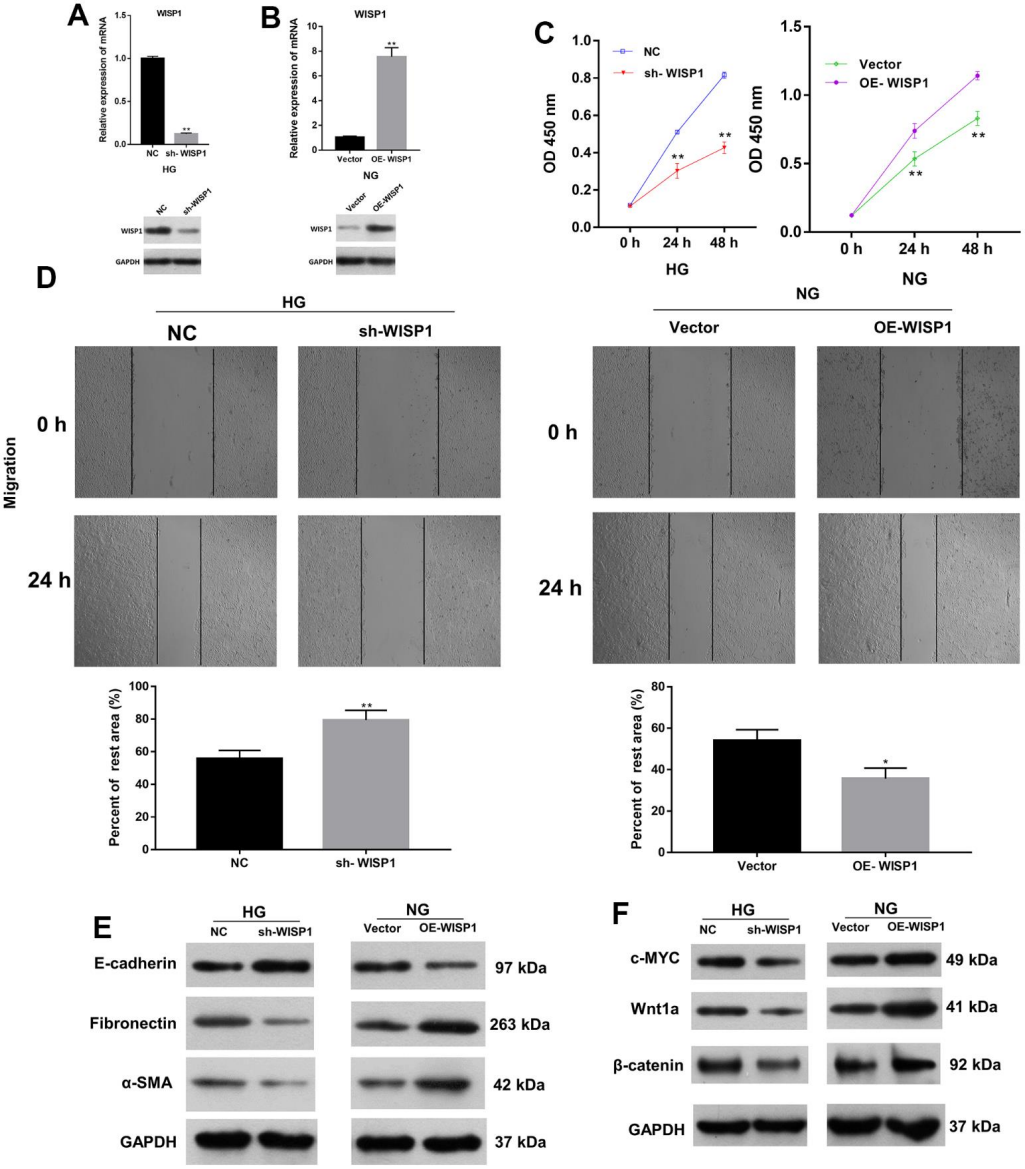
**The impacts of WISP1 on the proliferation, migration, EMT, and renal fibrosis of HG-treated HK2 cells.** (**A**) After HG treatment, the expression of WISP1 was confirmed using qRT-PCR and western blot in HK2 cells transfected with NC or sh-WISP1. (**B**) QRT-PCR and western blot analysis of WISP1 in HK2 cells transfected with OE-WISP1 or Vector. (**C**) CCK-8 assay for the assessment of cell proliferation in HG-induced HK2 cells with WISP1 silencing and HK2 cells with WISP1 overexpression. (**D**) Wound healing assay was conducted to identify the migration ability of the WISP1-silenced HG-induced HK2 cells and WISP1-overexpressed HK2 cells. (**E**) Western blot indicated the expression changes of E-cadherin, Fibronectin and a-SMA in WISP1-silenced HG-induced HK2 cells and WISP1-overexpressed HK2 cells. (**F**) The expressions of c-MYC, Wnt1 and β-catenin were monitored by applying western blot in each group. *P < 0.05, **P < 0.01.

On the basis of the above results, we ulteriorly verified the roles of WISP1 in HG-induced HK2 cells through a series of experiments. We first adopted qRT-PCR to evaluate the transfection effect of WISP1-silenced HG-induced HK2 cells and WISP1-overexpressed HK2 cells. As expected, in HG-induced HK2 cells, the level of WISP1 in the silencing group was prominently degraded relative to that in the NC group (Figure 2A). And in HK2 cells, the level of WISP1 in the overexpression group was memorably boosted relative to that in the vector group (Figure 2B). This implies that WISP1 has been successfully silenced in HG-induced HK2 cells or overexpressed in HK2 cells. Then the result of CCK-8 displayed that the proliferation of HG-treated HK2 cells was notably decreased in sh-WISP1 group in comparison with NC group, and the proliferation of HK2 cells was prominently enhanced in OE-WISP1 group compared to vector group (Figure 2C). Correspondingly, the result of wound healing exhibited that the migration ability of HG-treated HK2 cells dramatically decreased after WISP1 silencing, and upregulation of WISP1 observably promoted the migration ability of HK2 cells (Figure 2D). Furthermore, to investigate the relationship between EMT process and WISP1, we applied western blot to monitor the expression levels of EMT associated proteins. The results manifested that WISP1 silencing markedly upregulated E-cadherin, and downregulated a-SMA and Fibronectin in HG-treated HK2 cells, and WISP1 overexpression group had the opposite effects on the expression of these proteins in HK2 cells (Figure 2E). It is well known that EMT is typically characterized by suppression of E-cadherin, facilitation of Fibronectin and a-SMA expressions. So, we revealed that WISP1 overexpression could induce EMT of HK2 cells. Cell fibrosis is typically characterized by encouragement of Wnt1a, c-MYC and β-catenin expressions. Western blot results further disclosed that WISP1 silencing observably decreased c-MYC, Wnt1a, and β-catenin expressions in HG-treated HK2 cells, and WISP1 overexpression notably induced cell fibrosis in HK2 cells (Figure 2F). In short, we demonstrated that WISP1 could induce the development of DN.

### Increase of METTL3 expression memorably strengthened proliferation, EMT progression and fibrosis of HG-induced HK2 cells

**Figure 3:**
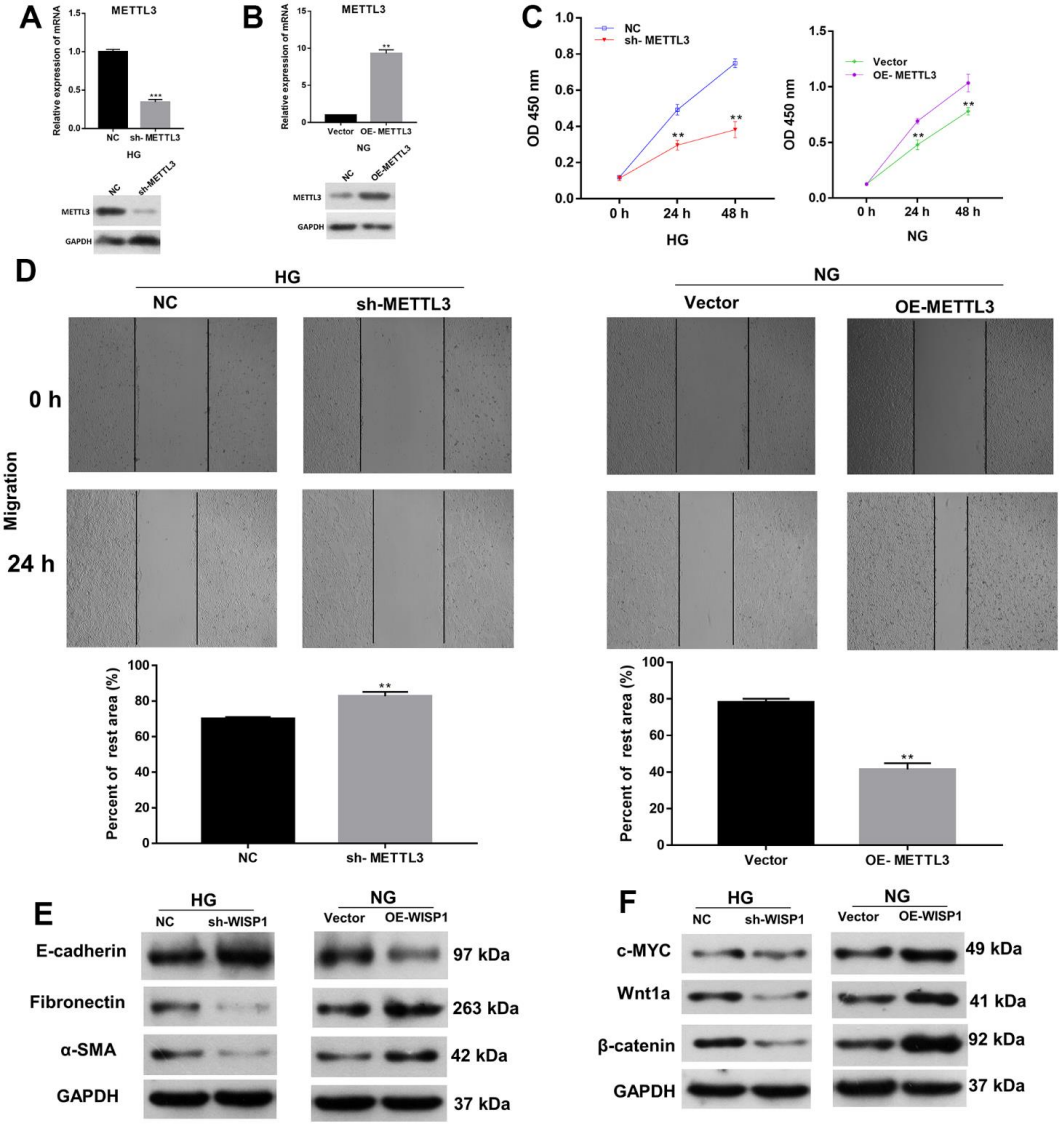
**Increase of METTL3 expression memorably strengthened EMT progression and fibrosis of HG-induced HK2 cells.** Sh-METTL3 or NC were transfected into HG-treated HK2 cells, and OE-METTL3 or vector were transfected into HK2 cells. (**A**) QRT-PCR and western blot analysis of METTL3 expression in METTL3-silenced HK2 cells after HG induction. (**B**) QRT-PCR and western blot analysis of METTL3 expression in METTL3-overexpressed HK2 cells. (**C**) CCK-8 assay demonstrated the change of cell proliferation. (**D**) The cell migration was credited through the application of wound healing assay. (**E**) The changes of E-cadherin, Fibronectin and a-SMA expressions were identified using western blot. (**F**) Western blot assay for the detection of c-MYC, Wnt1 and β-catenin expressions. **P < 0.01, ***P < 0.001.

Besides, we further investigated the roles of METTL3 in EMT and cell fibrosis progression of renal tubular epithelial cells. METTL3 was silenced in HG-treated HK2 cells and overexpressed in HK2 cells, respectively. Based on the qRT-PCR and western blot results, METTL3 was prominently downregulated in sh-METTL3 group, and upregulated in OE-METTL3 group (Figure 3A, 3B). Then we assessed the cell proliferation, and the results presented that the proliferation ability of HG-induced HK2 cells was memorably weakened in sh-METTL3 group relative to that in NC group, and the proliferation ability of HK2 cells was significantly enhanced in OE-METTL3 group relative to that in vector group (Figure 3C). Besides, wound healing results manifested that METTL3 silencing observably attenuated the migration of HG-treated HK2 cells, and overexpression of METTL markedly increased the migration of HK2 cells (Figure 3D). Meanwhile, western blot analysis of EMT and fibrosis marker proteins suggested that the upregulation of METTL3 worsened EMT and induced cell fibrosis in HG-induced HK2 cells, and downregulation of METTL3 had the opposite effect with METTL3 overexpression in HK2 cells (Figure 3E, 3F). Overall, these data indicated that METTL3 could accelerate cell proliferation, migration, EMT and fibrosis of HG-induced HK2 cells.

### Overexpression of WISP1 reversed the effects of METTL3 silencing on proliferation, EMT and fibrosis in HG-induced HK2 cells

**Figure 4:**
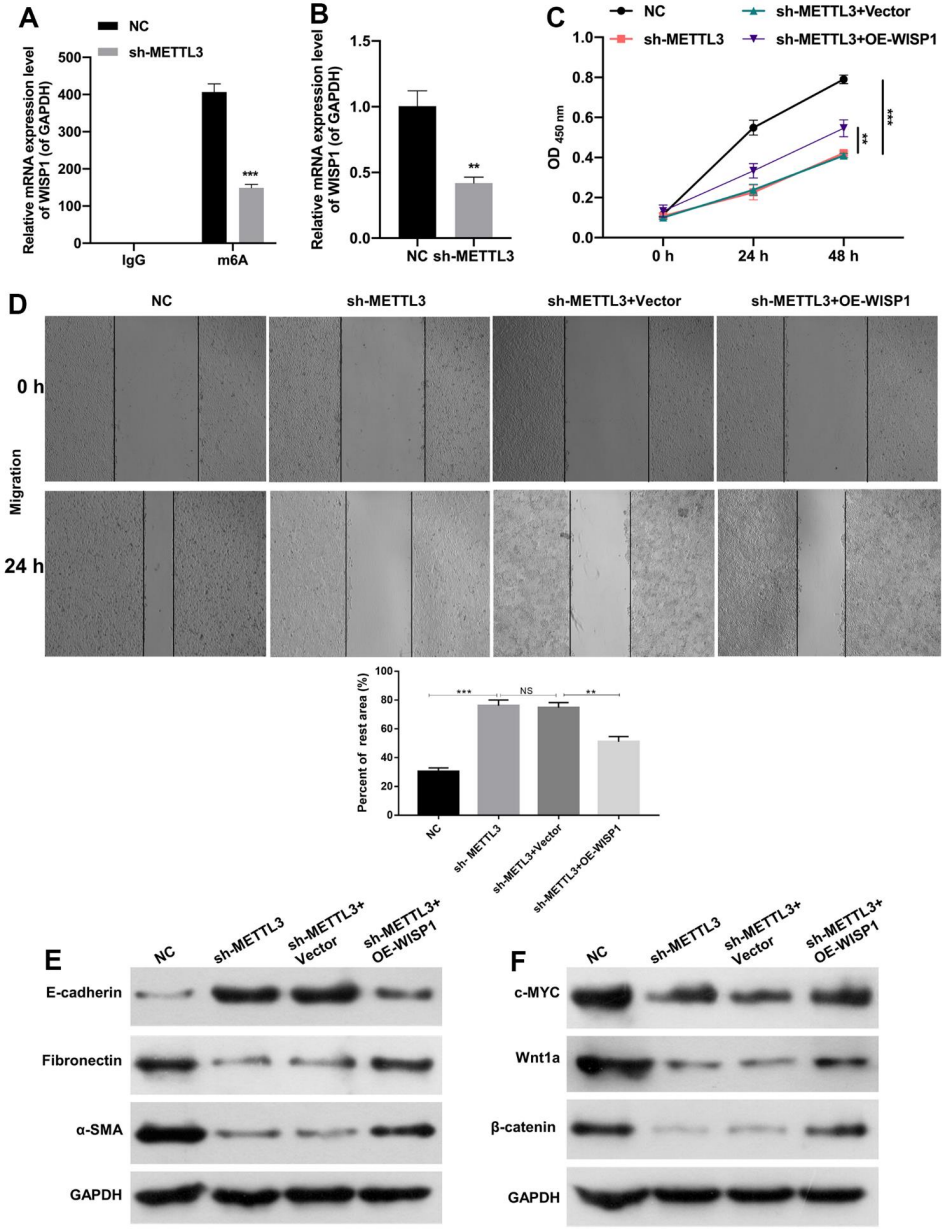
**METTL3 silencing prevented EMT progression and fibrosis of HG-induced HK2 cells by decreasing WISP1 with m6A modification.** (**A**) MeRIP-qPCR assay exhibited the change in WISP1 enrichment in HG-treated HK2 cells after METTL3 silencing. (**B**) WISP1 expression was examined via qRT-PCR in HG-treated HK2 cells which were transfected with METTL3 sh-RNAs. (**C**) HG-treated HK2 cells were transfected with sh-METTL3 or/and OE-WISP1, and the cell proliferation was tested by CCK-8 assay. (**D**) Wound healing assay showed the change of cell migration in the processed HK2 cells after HG treatment. (**E**) E-cadherin, Fibronectin and a-SMA expression levels were assessed by applying western blot. (**F**) Western blot was adopted to monitor the changes of c-MYC, Wnt1 and β-catenin expressions. **P < 0.01, ***P < 0.001.

Moreover, we further verified the moderating relationship between WISP1 and METTL3. And MeRIP-quantitative real-time PCR exhibited that WISP1 m^6^A modification was notably decreased after METTL3 silencing (Figure 4A). Then we discovered that METTL3 silencing also could notably downregulate WISP1 in HG-induced HK2 cells (Figure 4B). Next, we also confirmed the effects of WISP1 and METTL3 on cell-related functions by the rescue experiment. And HG-treated HK2 cells were co-transfected with sh-METTL3 and WISP1 overexpression plasmid. CCK-8 results displayed that silencing of METTL3 suppressed proliferation of HG-treated HK2 cells, while upregulation of WISP1 dramatically reversed the inhibition effect of METTL3 silencing on cell proliferation (Figure 4C). Similarly, the trend of cell migration was consistent with that of cell proliferation from CCK-8 results (Figure 4D). Besides, western blot results revealed that silencing of METTL3 dramatically upregulated E-cadherin, and downregulated a-SMA and Fibronectin in HG-treated HK2 cells, which also could be remarkably reversed by WISP1 overexpression (Figure 4E). Meanwhile, we testified that the downregulation of c-MYC, Wnt1a, and β-catenin mediated by METTL3 silencing also could be prominently restored by WISP1 overexpression in HG-treated HK2 cells (Figure 4F). Thus, we proved that METTL3 silencing could reduce WISP1 m^6^A modification, and silencing of METTL3 also could inhibit the degradation process of HG-treated HK2 cells by downregulating WISP1.

## DISCUSSION

DN is one of the major microvascular complications of diabetes, accounting for 30% to 40% of patients with diabetes [22]. DN has become the primary cause of end-stage renal disease worldwide and the principal cause of death in patients with diabetes [23]. DN is typically characterized by excessive deposition of extracellular matrix in the glomerulus and tubulointerstitium, ultimately causing glomerulosclerosis and tubulointerstitial fibrosis [24]. Study testified that glomerular injury is an early lesion of DN, and tubulointerstitial injury is secondary to glomerular injury [25]. Tubulointerstitial fibrosis usually occurs early in the course of diabetic kidney injury, which is associated with decreased renal function [26, 27]. Besides, the conversion of renal tubular epithelial cells to mesenchymal cells is one of the vital mechanisms of tubular interstitial fibrosis [28, 29]. Therefore, further investigation of renal tubular epithelial cell EMT and renal fibrosis is of crucial importance for DN therapy.

The severity of chronic kidney disease (CKD) is positively correlated with the degree of kidney fibrosis, causing end-stage renal disease [30]. The Wnt/β-catenin pathway plays a key role in organ development, tissue homeostasis and injury repair [31]. This pathway is relatively silent in normal adult kidneys, but can be reactivated in uremic model animals and in patients with CKD [32]. Research demonstrated that the Wnt/β-catenin pathway can mediate myofibroblast activation, proliferation and EMT in renal tubular epithelial cells by affecting the expression of related target genes such as fibronectin and E-calcineurin, leading to renal fibrosis [18]. And WISP1 is a key downstream molecule of the Wnt/β-catenin pathway [19]. WISP1 has been reported to be associated with pathological processes such as inflammation, injury repair, and tumor development [20, 21, 33]. Additionally, WISP1 expression can be increased in renal injury models, which may promote the development of renal fibrosis and accelerate the process of CKD [34]. While the role of WISP1 in DN is not fully understood. In our study, we further proved that WISP1 was highly expressed in renal tissues of DN model mice. Meanwhile, we verified that WISP1 silencing could weaken the proliferation, migration, and fibrosis of HG-induced HK2 cells, and WISP1 overexpression could enhance the proliferation, migration, and fibrosis of HK2 cells.

EMT is an evolutionary conserved biological process of epithelial to mesenchymal cell transformation [35]. The EMT phenotypic state is highly dynamic and dependent on cell type and environment [36]. Therefore, the definition of EMT should be evaluated in the context of cellular characteristics and multiple molecular markers, including α-SMA, E-cadherin and fibronectin [37, 38]. In our study, we also revealed that WISP1 silencing can prevent EMT of HG-treated HK2 cells, and WISP1 overexpression can accelerate EMT of HK2 cells through the detection of related molecules.

M^6^A methylation modification is the most widespread RNA epigenetic modification in eukaryotes [39]. M^6^A modification is a dynamically reversible post-transcriptional modification that is most abundant in mRNAs and non-coding RNAs [40]. And m^6^A plays a key role in gene expression and cell fate regulation [41]. In recent years, several studies have confirmed the essential role of m^6^A modification in DN [42–44]. METTL3, as a major methyltransferase for m^6^A methylation, has a key role in cell proliferation, differentiation, metastasis and other cellular biological processes [45, 46]. However, only two papers have reported the role and mechanism of METTL3 in DN [14, 15]. In our study, we further confirmed that METTL3 silencing could suppress the proliferation, EMT, migration, and fibrosis of HG-treated HK2 cells, METTL3 overexpression could induce the proliferation, EMT, migration, and fibrosis of HK2 cells. Besides, we first discovered that METTL3 silencing could reduce WISP1 m6A modification, suggesting METTL3 could mediate m6A modification of WISP1 mRNA. More importantly, we uncovered that METTL3 silencing could prevent DN process by reducing WISP1 mediated by m6A modification.

In summary, METTL3 plays a key role in human kidney proximal tubular epithelial cell proliferation, EMT and fibrosis, and knockdown of METTL3 could impair EMT and fibrosis in HG-treated HK2 cells. Molecularly, METTL3 silencing could reduce WISP1 transcript level by attenuating WISP1 mRNA m^6^ methylation modification. And METTL3 silencing also could alleviate DN progression by reducing WISP1 with m6A modification. Therefore, targeted inhibition of METTL3/WISP1 axis is expected to be a key molecular target for DN intervention.

## MATERIALS AND METHODS

### Experimental animals

A total of 10 specific Pathogen Free (SPF) grade C57/BL6 mice (male, 8 weeks old, weight 20-25 g) were purchased from the Animal center of Sun Yat-Sen University. Feeding conditions:12 h light day and night, 22-24° C, 40%-70% humidity, well ventilated, ad libitum diet. Animal experiments were performed under the institutional guidelines of the Animal Care and Use Committee of University of Chinese Academy of Sciences Shenzhen Hospital.

### Construction of DN model mice

Ten mice were randomly divided into control group and DN model group, with 5 mice in each group. The mice in the model group were fed with high-fat diet for 4 weeks. Subsequently, the diabetic mouse model was established by multiple small-dose intraperitoneal injections of streptozotocin (40 mg/kg for 5 d), and the mice in the control group were injected intraperitoneally with an equal amount of saline. Monitoring of blood glucose concentration was started 72 h after the completion of injection, and the modeling of diabetes was considered successful if the random blood glucose concentration was >16.7 mmol/L. Thereafter, blood glucose and weight were measured regularly once a week, and a 24-h urine protein level >30 mg after 4 weeks indicated successful diabetic nephropathy modeling.

### Cell culture

Human kidney proximal tubular epithelial cells (HK2) were from BeNa Culture Collection Company (Beijing, China). After resuscitation, HK2 cells were centrifuged to collect cells. then HK2 cells were grown in DMEM (Aidenbach; Germany) containing 10% fetal bovine serum (FBS, Gibco, USA) at 37° C with 5% CO_2_. The DMEM medium containing 5.6 mM glucose was set as normal-glucose (NG) group, while the medium containing 30 mM glucose were set as high-glucose (HG) group.

### Cell treatment

Negative control (NC), WISP1 shRNAs (sh-WISP1), METTL3 shRNAs (sh-METTL3), vector, WISP1 overexpression plasmid (OE-WISP1), METTL3 overexpression plasmid (OE-METTL3) were provided by Integrated Biotech Solutions (Shanghai, China). HK2 cells at logarithmic growth stage were seeded in 96-well plates at 3×10^5^ cells/well and treated with HG for 48 h. Then HG-treated HK2 cells were transfected with shRNAs or NC, HK2 cells were transfected with the overexpression plasmids or vector for 48 h using Lipofectamine 3000 (Invitrogen, USA) based on the instructions.

### Quantitative real-time PCR (qRT-PCR)

Total RNA from the ground kidney tissue or the treated HK2 cells was extracted using Trizol Reagent. After purity and concentration were assessed, the RNA was reverse transcribed to acquire cDNA using PrimeScript™ RT reagent kits (Takara, Dalian, China). Then qRT-PCR was conducted with 2×SYBR Green qPCR Master Mix (Applied Biosystems, USA). The relative levels of METTL3 and WISP1 were calculated by 2^-ΔΔCt^ method.

### CCK-8

The processed HK2 cells were inoculated in 96-well plates with 5×10^3^ cells/well and incubated at 37° C. At 0, 24 and 48 h, each well was added with 10 μL CCK-8 solution (Dojindo, Kumamoto, Japan) and fostered in the incubator for 1h. And the absorbance value at 450 nm was carried out by microplate reader.

### Wound healing

The processed HK2 cells (5×10^4^ cells) were evenly inoculated in 6-well plates. When the cells showed adnate growth, they were scratched. Then the detached cells were washed off with PBS and the adherent cells were added with serum-free culture medium. After 24 h of continued incubation, the width of the scratch was observed and recorded under a light microscope.

### Western blot

The total protein of HK2 cells was extracted using RIPA lysis buffer (Beyotime, Shanghai, China) with 1% PMSF (Beyotime). Then the protein concentration was monitored through BCA kit (Beyotime). 40 μg of protein was subjected to SDS-PAGE gel electrophoresis and transmembrane with 0.22 μm PVDF membrane. After blocking, the membranes were processed with the diluted primary antibodies (Abcam, Cambridge, UK) at 4° C overnight and secondary antibody (Abcam) for 2 h. After treating with Pierce™ ECL substrate (Thermo Fisher Scientific, USA), the blots were colored with an ECL system (Thermo Fisher Scientific).

### MeRIP-quantitative real-time PCR

20 μg of total RNA was processed with DNAase I and the RNA free of DNA contamination was digested with RNA fragmentation reagent at 90° C for 30 s. After termination of the reaction, the fragmented RNA was re-extracted by ethanol sedimentation. Subsequently, 2 μg of m6A antibody was co-incubated with 40 μl ProteinA/G magnetic beads in IPP buffer for 1 h. The fragmented RNA was added to the above system and incubation was continued at 4° C for 4 h. After washing the magnetic beads 4 times with IPP buffer, the samples were added to Trizol to extract RNA from the magnetic beads. Finally, qRT-PCR analysis was conducted.

### m6A RNA methylation assay

The total amount of m6A in total RNA was measured using the m6A RNA Methylation Assay Kit (Abcam), following the manufacturer manual. 200 ng total RNA from renal tissues or cells was used.

### Statistical analysis

All data were processed using SPSS 23.0 software (SPSS Inc., USA), and the measurement data were signified as mean ±SD. One-way ANOVA was adopted for comparison between multiple groups, and t-test was adopted for comparison between two groups. And the difference was defined as statistically significant at *P* < 0.05.
